# Probing higher order optical modes in all-dielectric nanodisk, -square, and -triangle by aperture type scanning near-field optical microscopy

**DOI:** 10.1515/nanoph-2021-0612

**Published:** 2021-12-22

**Authors:** Aleksandr Yu. Frolov, Joris Van de Vondel, Vladimir I. Panov, Pol Van Dorpe, Andrey A. Fedyanin, Victor V. Moshchalkov, Niels Verellen

**Affiliations:** Faculty of Physics, Lomonosov Moscow State University, Moscow, Russian Federation; Department of Physics and Astronomy, Quantum Solid-State Physics, KU Leuven, Leuven, Belgium; imec, Leuven, Belgium; Department of Physics and Astronomy, Quantum Solid-State Physics, KU Leuven, Leuven, Belgium

**Keywords:** all-dielectric nanophotonics, cavity and whispering gallery modes, higher order modes, nanoantenna, odd and even symmetry, scanning near-field optical microscopy

## Abstract

All-dielectric nanoantennas, consisting of high refractive index semiconductor material, are drawing a great deal of attention in nanophotonics. Owing to their ability to manipulate efficiently the flow of light within sub-wavelength volumes, they have become the building blocks of a wide range of new photonic metamaterials and devices. The interaction of the antenna with light is largely governed by its size, geometry, and the symmetry of the multitude of optical cavity modes it supports. Already for simple antenna shapes, unraveling the full modal spectrum using conventional far-field techniques is nearly impossible due to the spatial and spectral overlap of the modes and their symmetry mismatch with incident radiation fields. This limitation can be circumvented by using localized excitation of the antenna. Here, we report on the experimental near-field probing of optical higher order cavity modes (CMs) and whispering gallery modes (WGMs) in amorphous silicon nanoantennas with simple, but fundamental, geometrical shapes of decreasing rotational symmetry: a disk, square, and triangle. Tapping into the near-field using an aperture type scanning near-field optical microscope (SNOM) opens a window on a rich variety of optical patterns resulting from the local excitation of antenna modes of different order with even and odd parity. Numerical analysis of the antenna and SNOM probe interaction shows how the near-field patterns reveal the node positions of – and allows us to distinguish between – cavity and whispering gallery modes. As such, this study contributes to a richer and deeper characterization of the structure of light in confined nanosystems, and their impact on the structuring of the light fields they generate.

## 1 Introduction

All-dielectric nanostructures are becoming key elements in modern nanophotonics R&D, allowing efficient control of the phase, polarization, and direction of light [1]. Two-dimensional arrangements of subwavelength nanoparticles make it possible to create ultrathin metasurfaces [2] operating as efficient lenses [3], polarizers [4], non-linear [5–9] and magneto-optical devices [10, 11], ultrafast switches [12, 13], light emitting devices [14–16], directional light scatterers [17–20], and photonic topological insulators [21, 22]. A single all-dielectric nanoparticle in such metasurfaces operates as an optical nanoantenna that manipulates light through the excitation of various electric and magnetic optical modes [23].

The full mode spectrum of a nanoantenna can be significantly expanded by giving it a non-spherical shape. It enables the excitation of higher order modes and thereby offers opportunities for more elaborate light flow manipulation and tunability compared to low order modes. Depending on the spatial field distribution, the higher order modes are localized either inside the whole volume of the nanoantenna – cavity modes (CMs), or around the nanoantenna’s edge – whispering gallery modes (WGMs). Spatial and spectral overlapping of higher order CMs and WGMs in nanoantennas complicates their analysis using conventional far-field optical techniques. Moreover, the presence of mirror symmetry planes of complex shaped nanoantennas splits optical modes in ones with even or odd parity (symmetry). As the electromagnetic field of a plane wave source at normal incidence possesses even and odd parity with respect to the two symmetry planes, it allows coupling with only specific modes [24–28]. Whereas modes with mismatching symmetry are forbidden for excitation, inclined illumination is able to break this symmetry coupling rule [29–32]. Numerical works have shown, nevertheless, that a point dipole source can efficiently couple to both even and odd parity modes and as such modify its emission [33–35]. Therefore, local excitation within the near-field becomes a crucial approach to excite and visualize higher order modes in all-dielectric nanoantennas of increasingly complex shapes.

Various optical techniques exist for the local excitation and visualization of optical modes in photonic structures at the nanoscale. The visualization of plasmonic charge density waves in gold microstructures was realized by scanning a plasmonic antenna with a highly focused light beam while recording the scattered intensity [36]. Angular-resolved cathodoluminescence using a high energy electron beam allows the local excitation and mapping of high and low order radial and azimuthal resonant modes in silicon (Si) nanodisks [37], and hybridized symmetric and antisymmetric modes in Si bar dimers [38]. A wide variety of even and odd surface plasmon modes have been mapped using electron beams in metallic nanoantennas of different shapes: rod [39], disk [40], square [41], triangle [42], and their coupled systems [43–45].

Alternatively, the mapping of optical mode distributions with subwavelength resolution can be achieved using scanning near-field optical microscopy (SNOM). Here, either a sharp metal coated needle-like (apertureless) probe or a hollow, metal coated probe with a subwavelength hole at its apex (aperture) is scanned over the surface of the nanophotonic systems. An SNOM measurement is typically performed with one of two conventional approaches: the collection or illumination scheme.

In the collection scheme, the modes of the nanoantennas are excited from the far-field, and an aperture or apertureless probe picks up their near-field. Such near-field studies have been performed on plasmonic nanoantennas of different shapes including disks, rods, triangles, Yagi-Uda antennas [46–51] and all-dielectric nanoantennas with, e.g., the visualization of electric quadrupole [52] and anapole [53, 54] modes in Si-nanodisks, and the magnetic field in the gap of Si-nanodisk dimers [55]. However, such a technique allows coupling only to a limited number of modes available for plane wave excitation, and tilted illumination should be used in order to access both even and odd parity modes.

In the SNOM illumination scheme, a localized near-field light source is generated around the apex of an aperture or apertureless probe by illuminating it from the far-field. In this case, we obtain a unique subwavelength polarized light source. This localized source can provide a symmetry breaking effect that allows an efficient excitation of even and odd parity modes. As such, multiple plasmonic modes in various antenna shapes [56–59] and optical Fabry–Pérot modes with odd and even parity in amorphous silicon nanorods [60] have been mapped. Notwithstanding that such a variety of higher order optical modes has been observed in plasmonic structures of various shapes, near-field probing of all-dielectric nanoantennas was performed only in disk and rod shapes where several higher order spectrally and spatially separated optical modes have been studied. The modification of a dielectric antenna shape expands the set of higher-order CMs and WGMs with high *Q*-factor. The spectral and spatial overlapping of CMs and WGMs can be exploited to increase the optical density of states which is crucial for light-emitting and non-linear devices. However, the coexistence of multiple spectrally overlapped higher order modes at a single wavelength significantly complicates the analysis and interpretation of SNOM data as compared to a single optical mode.

In the current work, we used the aperture type scanning near-field optical microscopy for the probing of higher CMs and WGMs in all-dielectric nanoantennas with basic geometries of different rotational symmetry. We show experimentally and numerically that the SNOM aperture probe in the illumination scheme excites higher order transverse electric and magnetic modes in disk, square, and triangular antennas consisting of amorphous silicon (*α*-Si). Due to the coexistence of a rich variety of modes at a single wavelength, the SNOM maps do not necessarily represent the field distribution of individual optical modes but rather a superposition. Therefore, to provide a thorough analysis of the intensity contrast in SNOM maps, the scanning process of each nanoantenna was simulated with a finite-difference time-domain (FDTD) solver. From these simulations, we can expose the coincidence of features in the SNOM maps and the antennas’ electric or magnetic field modal distributions. At the same time, the wavelengths and probe positions that result in resonant excitation of the different modes can be identified by maxima of the electric field localization.

## 2 Results and discussion

**Figure 1:**
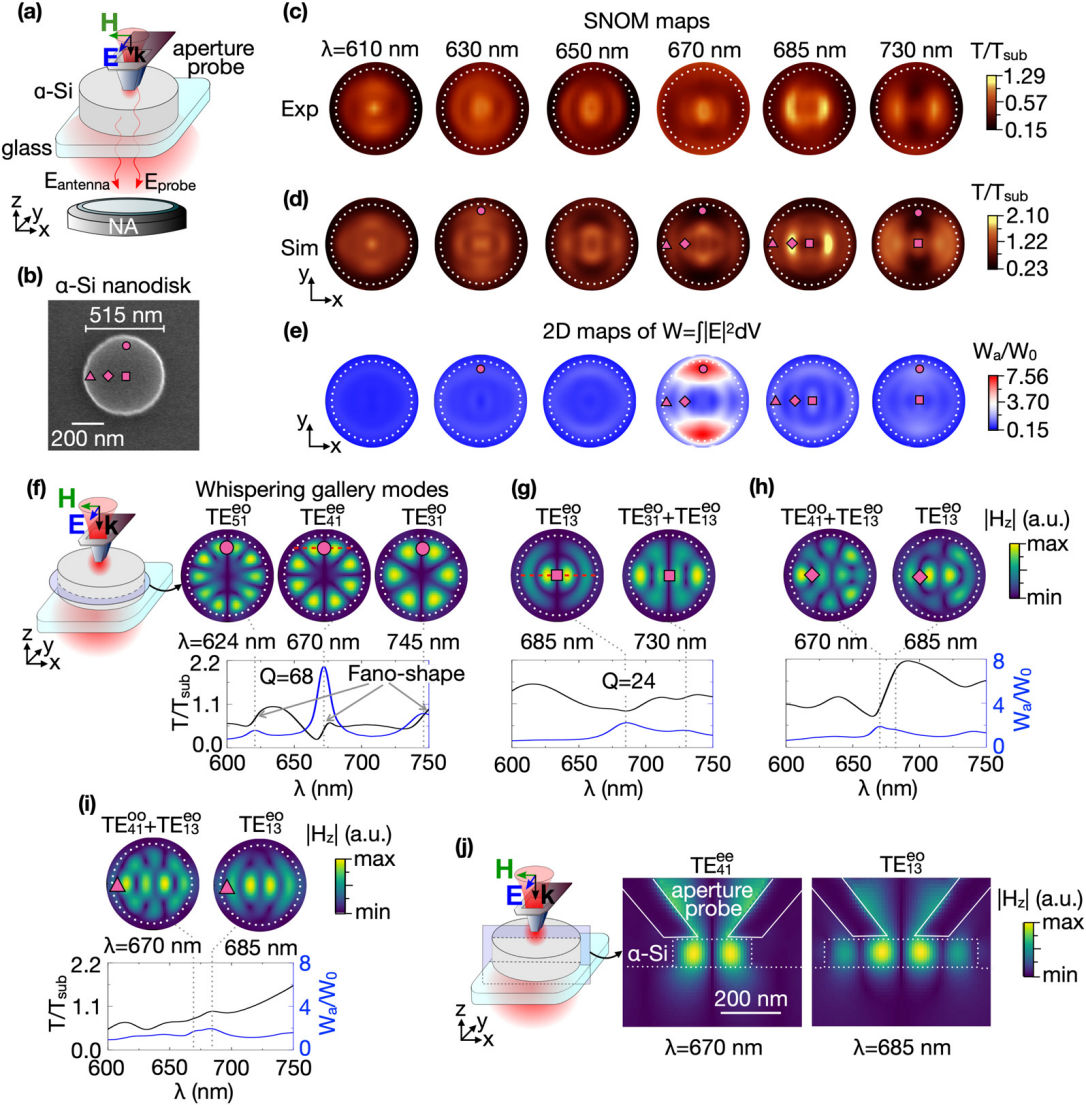
(a) Schematic representation of the near-field mapping of optical modes in the *α*-Si nanodisk. (b) SEM image of the *α*-Si nanodisk. The diameter is 515 nm, and the thickness is 96 nm. Experimental (c) and simulated (d) SNOM maps of the *α*-Si nanodisk at different wavelengths. The polarization of light incident on the SNOM probe (blue arrow) is directed along the *y*-axis. (e) Simulated 2D maps of electric field localization W_*a*_/W_0_ at the same wavelengths as in the SNOM maps in (c) and (d). The circle (*x* = 0 nm, *y* = 220 nm), square (*x* = 0 nm, *y* = 0 nm), diamond (*x* = 120 nm, *y* = 0 nm), and triangle (*x* = 240 nm, *y* = 0 nm) symbols on 2D maps in (b), (d), and (e) depict the SNOM probe positions corresponding to maxima of the electric field localization W_*a*_/W_0_. (f–i) The simulated SNOM transmittance *T*/*T*_sub_ (black curves) and electric field localization W_*a*_/W_0_ (blue curves) spectra recorded at the fixed positions of the SNOM probe marked as purple symbols on SNOM (d) and W_*a*_/W_0_ (e) maps. The top row in (f–i) shows the simulated |*H*_*z*_| distributions of ${TE}_{mn}^{ij}$ optical modes. The |*H*_*z*_| field was taken at central *xy*-cross-sections of the nanodisk as shown in the sketch in panel (f). (j) |*H*_*z*_| distributions of ${TE}_{41}^{ee}$ and ${TE}_{13}^{eo}$ optical modes at *xz*-cross-section (see schematic illustration in the sketch). The positions of *xz*-plane cuts are marked by red dashed lines in (f) and (g). Dotted white circles indicate the boundary of the nanodisk. The solid white lines indicate the edges of the aperture SNOM probe.

The experimental scheme for the near-field mapping is illustrated in Figure 1(a). The local excitation of the optical modes is obtained by focusing polarized supercontinuum laser light in the wavelength range from *λ* = 600 nm to *λ* = 750 nm on a subwavelength hole at the apex of 100 nm Al-coated aperture SNOM probe (see the Measurements and simulations in Methods section, Supplementary Materials for more details). The full SNOM map of each nanoantenna is obtained by scanning its surface with the probe in contact mode and recording the transmitted light intensity (*T*) collected by the objective lens shown at the bottom.

### 2.1 Disk

We start the discussion with the study of an *α*-Si nanodisk on a glass substrate. The disk diameter is 515 nm and thickness is 96 nm (see the SEM image in Figure 1(b)). For details on the sample fabrication, we refer to the sample fabrication in Methods section and atomic force microscopy section in Supplementary Materials. Experimental SNOM maps at different wavelengths in the range from *λ* = 600 nm to *λ* = 750 nm are shown in Figure 1(c). SNOM maps consist of the bright and dark areas corresponding to high and low light transmittance through the SNOM probe–antenna system. The transmittance is modified due to the excitation of higher order optical modes in the nanodisk. The strong wavelength dependence of the patterns in these SNOM maps is similar to the frequency dependence of acoustic Chladni patterns observed on a metallic plate covered by sand and locally driven by a bow [61]. Using full-field 3D FDTD simulations, the experimental maps can accurately be reproduced, as is demonstrated by comparing panel (c) with (d) in Figure 1. The full scanning near-field microscopy configuration is taken into account for these simulations, including the nanoantenna, the substrate, and the probe (see SNOM simulations in the Methods section, Supplementary Materials for more details). To show how the excited antenna modes modify the probe transmittance *T*, the color scale is normalized to the transmittance at the glass substrate, *T*_sub_, recorded at 3 μm distance from the disk boundary. The nanodisk resonator with high aspect ratio supports a rich variety of coexisting optical modes at the same wavelength. Determining the order and type of the excited modes requires figuring out the probe positions and wavelengths at which resonant excitation occurs. To this end, we calculated the integral of the electric field localized inside the antenna volume, W_*a*_ = ∫|*E*|^2^d*V*, recorded at each position of the SNOM probe on the nanoantenna (see SNOM simulations in the Methods section, Supplementary Materials). The spectral dependence of 2D maps of W_*a*_ normalized to the electric field localization without antenna W_0_ is presented in Figure 1(e). The maxima on these W_*a*_ */*W_0_ maps are located both inside the disk and near its edges. For *λ* = 610 nm they are negligible, while for longer wavelengths from *λ* = 630 nm to *λ* = 730 nm, they are clearly distinguished. The circle, square, diamond, and triangle purple symbols in Figure 1(e) mark the prominent W_*a*_/W_0_ maxima located near and far from nanodisk’s edge. The near-field distributions of the optical modes shown in the top row of panels (f–i) present FDTD simulations of the dominant field component along *z*-direction at the wavelengths corresponding to W_*a*_/W_0_ maxima. These wavelengths were identified from the spectral dependence of the field localization W_*a*_/W_0_ (blue curves in panels (f–i)) recorded at the probe positions marked with the purple symbols on the 2D SNOM (panel (d)) and W_*a*_/W_0_ (panel (e)) maps. To categorize the different optical modes supported by the dielectric nanodisk cavity, we follow the nomenclature of microwave dielectric cylindrical resonator antennas and waveguides [63]. ${TE}_{mn}^{ij}$ designates a hybrid mode which has the |*H*_*z*_| component as the dominant contribution to the total |**H**| field distribution. The subscripts *m* and *n* refer to the waveguide mode designation, where *m* indicates the total number of wavelengths that fit the disk periphery in the azimuthal direction (i.e., half of the antinodes number fitted along the periphery) and *n* is the number of antinodes fitted along the radial direction. We verify that only one |*H*_*z*_| antinode exists in the *z*-direction for the observed modes, i.e., only half of an effective wavelength fits within the antenna thickness. The superscripts *i* = *e*, *o* and *j* = *e*, *o* designate the even (*e*) or odd (*o*) mode parity relative to the antenna’s mirror symmetry plane perpendicular to the *x*-axis (*σ*_*x*_-plane) and *y*-axis (*σ*_*y*_-plane), respectively. Since the magnetic field **H** is a pseudovector, the *H*_*z*_ component of even parity modes transforms as an antisymmetric function (*H*_*z*_(*x*, *y*) = −*H*_*z*_(−*x*, *y*), *H*_*z*_(*x*, *y*)) = −*H*_*z*_(*x*, −*y*)) under mirror reflections with respect to *σ*_*x*_ and *σ*_*y*_ planes, and as a symmetric function (*H*_*z*_(*x*, *y*) = *H*_*z*_(−*x*, *y*), *H*_*z*_(*x*, *y*) = *H*_*z*_(*x*, −*y*)) in the case of odd parity modes.

When the probe locates near the edge of the disk (circle symbol marking in Figure 1(f)), the W_*a*_/W_0_ spectrum has three maxima which correspond to the excitation of the following WGMs: ${TE}_{51}^{eo}$ at *λ* = 624 nm, ${TE}_{41}^{ee}$ at *λ* = 670 nm, and ${TE}_{31}^{eo}$ at *λ* = 745 nm. It is also observed that the SNOM probe positions, that result in resonant excitation, coincide with the |*H*_*z*_| nodes of these WGMs.

Next, we discuss the optical modes excited at other probe positions. When the probe locates in the disk center (square mark in Figure 1(g)), the type of modes changes from WGMs, which are mostly concentrated near the disk edge, to CMs, which cover the full disk volume. The maximum at *λ* = 685 nm of the corresponding W_*a*_/W_0_ spectrum is associated with the ${TE}_{13}^{eo}$ mode (*σ*_*x*_-even, *σ*_*y*_-odd). Due to the arrangement of three |*H*_*z*_| antinodes along the *x*-axis (counting from the center of the disk), the ${TE}_{13}^{eo}$ mode is again excited by the SNOM probe at the two off-center positions marked as diamond and triangle in Figure 1(h) and (i), respectively. These positions also coincide with |*H*_*z*_| nodes of the ${TE}_{13}^{eo}$ mode. In addition to the excitation of individual modes, Figure 1(g) shows that the probe simultaneously excites a superposition of modes at the square mark position at *λ* = 730 nm (superposition of ${TE}_{31}^{eo}$ and ${TE}_{13}^{eo}$ modes). The superposition of ${TE}_{41}^{oo}$ and ${TE}_{13}^{eo}$ modes is observed at the positions marked as diamond (panel (h)) and triangle (panel (i)) at *λ* = 670 nm. This simultaneous excitation occurs when the individual modal antinodes and spectral bands overlap (see Section 3, Supplementary Materials with the decomposition of the *H*_*z*_ field distribution in the individual eigenmodes).

The positions of the SNOM probe, at which the resonant excitation takes place, coincide with nodes of the |*H*_*z*_| distribution for all excited optical modes. Figure 1(j) shows in more detail the coupling of the probe near-field with ${TE}_{41}^{ee}$ and ${TE}_{13}^{eo}$ modes. It presents the simulated |*H*_*z*_| field distribution in the *xz*-plane (see sketch in (j)) taken through the circle and square probe positions along the red dashed lines in (f) and (g). The SNOM probe near-field, created by the incident focused laser light with linear polarization, possesses two strong |*H*_*z*_| hotspots (see also field distribution of all components near the aperture SNOM probe in Supporting Information in Ref. 56). At the position of the modal |*H*_*z*_| nodes, the two magnetic field hotspots of the probe apex spatially overlap with two |*H*_*z*_| antinodes of ${TE}_{41}^{ee}$ and ${TE}_{13}^{eo}$ modes resulting in the resonant excitation. The reason for the efficient excitation of optical modes at the node of the normal field components is the absence of the normal magnetic field component at the center of the SNOM probe [56, 62]. In contrast to the zero normal magnetic component, a strong lateral electric field component (along the *y*-axis) exists at the center of the SNOM probe. Therefore, the resonant position also corresponds to the antinodes of the lateral electric field. Similar correspondence of lateral field antinodes with resonant position of the SNOM probe has been observed for plasmonic [56, 58] and all-dielectric nanoantennas [60].

Now we discuss the transmission intensity contrast that is observed in the SNOM maps. For this, we focus on the most prominent W_*a*_/W_0_ maxima positions in Figure 1(e) and superimpose them on the simulated SNOM maps in Figure 1(d) (purple marks). As such, we can associate the excitation of specific optical modes with the resulting transmittance features. A quick look at Figure 1(d) and (e) already reveals that there is no straightforward one-to-one correlation between the excitation of a mode and the SNOM contrast. Indeed, the maxima of the field localization can coincide with both local dark or bright spots, or even somewhere in between. This leads us to conclude that the SNOM intensity contrast is the result of a more intricate interplay between multiple coexisting optical modes that can be excited at the same wavelength. The scattering intensity of each optical mode can be high or low, which results in bright or dark spots, respectively. For the SNOM map at *λ* = 630 nm (Figure 1(d)), ${TE}_{51}^{eo}$ WGM excitation results in the bright spot. The other *T*/*T*_sub_ maxima and minima on this SNOM map near the disk center are also associated with the excitation of other higher order modes. However, their electric field localization is lower than that of ${TE}_{51}^{eo}$ . For the SNOM map at *λ* = 685 nm, ${TE}_{41}^{ee}$ WGM excitation leads to the dark spot (circle in Figure 1(d)), while the ${TE}_{41}^{oo}+{TE}_{13}^{eo}$ mode appears as local bright spots at the diamond and triangle dots. For the SNOM map at *λ* = 685 nm, the excitation of the ${TE}_{13}^{eo}$ mode at their three |*H*_*z*_| nodes (square, diamond, and triangle) results in three bright spots with various *T*/*T*_sub_ values of 0.9, 2.1, 0.97, respectively. Finally, for the SNOM map at *λ* = 730 nm, the ${TE}_{31}^{eo}$ WGM appears as the local dark spot (circle mark), but the ${TE}_{13}^{eo}+{TE}_{31}^{eo}$ mode leads to the bright spot (square mark).

Figure 1(g)–(i) depicts the spectral dependence of the transmittance *T*/*T*_sub_ (black curves) and field localization W_*a*_/W_0_ (blue curves), for fixed positions of the SNOM probe as indicated with the purple dots in Figure 1(d) and (e). Also here, it is seen that there is no straightforward link between the local excitation of a mode and the resulting *T*/*T*_sub_ behavior, i.e., there is no spectral match between peaks and dips. A closer look, however, reveals that the *T*/*T*_sub_ curves possess asymmetric Fano lineshapes in the spectral vicinity of resonant modes excitation (the W_*a*_/W_0_ maxima). The asymmetric lineshape arises from the interference of the two radiative contributions reaching the objective: the first one being the resonant radiation of an optical mode (*E*_antenna_, see Figure 1(a)) and the second being light non-resonantly transmitted through the antenna (*E*_probe_, see Figure 1(a)). The latter comes from propagating modes of the illuminated probe [64]. Similar spectral behavior of the transmittance spectrum (Fano lineshape) has been observed for plasmonic nanoantennas probed by aperture type SNOM in the illumination mode [65, 66]. The *T*/*T*_sub_ minima (maxima), which are blue-(red-) shifted from the W_*a*_/W_0_ maxima in Figure 1(f–i), are the results of the destructive (constructive) interference between the antenna modes and propagating modes of the probe. Consequently, this Fano-interference, along with various scattering intensities of each mode, influences the SNOM map intensity contrast. Each mode can manifest itself as a bright or dark spot depending on the observation wavelength and the transmittance of other coexisting modes. Moreover, the SNOM probe position determines the transmittance properties of the individually excited mode. It is seen from the excitation of the ${TE}_{13}^{eo}$ mode at its three |*H*_*z*_| nodes (square, diamond, and triangle symbols in panel (d) at *λ* = 685 nm) where three bright spots correspond to different *T*/*T*_sub_ values of 0.9, 2.1, and 0.97, respectively. The corresponding electric field localization values are 2.27, 1.49, 1.92, and correlate oppositely with the *T*/*T*_sub_ values. W_*a*_/W_0_ at the second |*H*_*z*_| node (diamond, Figure 1(h)) is lower than at two other nodes (square and triangle in Figure 1(g) and (i), respectively). The lower W_*a*_/W_0_ at the second |*H*_*z*_| node means lower coupling efficiency of the probe near-field with the ${TE}_{13}^{eo}$ mode resulting in higher non-resonant scattering intensity from the SNOM probe and therefore a brighter spot on the SNOM maps. The coupling efficiency at the second |*H*_*z*_| node (diamond mark) is lower as the SNOM probe near-field is coupled with two |*H*_*z*_| antinodes of different spatial shapes (one circular and one half-moon shaped) in comparison with ones at the center and edge positions (see |*H*_*z*_| distribution of ${TE}_{13}^{eo}$ mode in panels (g, h, i)). Hence, the *T*/*T*_sub_ contrast of an individual excited optical mode is also modified when the probe drives the same mode at its different |*H*_*z*_| nodes. An additional contribution to the modification of the *T*/*T*_sub_ value, appearing when the same mode is excited at its different nodes, is the dependence of the phase shift between resonant and non-resonant radiation on the relative position of the SNOM probe and the nanoantenna’s center. The phase shift is modified when the probe excites the same mode at its nodes which are closer or further from the antenna’s center.

Compared with other works on SNOM near-field mapping, the observed SNOM contrast for the *α*-Si nanodisk is different from the contrast observed for the excitation of the spectrally and spatially separated Fabry–Perot modes in all-dielectric [60] and plasmonic 1D nanorod [56, 67] antennas. In those nanoantennas, the bright (for dielectric nanorod) and dark (for plasmonic nanorod) spots on the SNOM maps coincide with the probe positions of resonant excitation. However, similar to the obtained SNOM maps for a *α*-Si nanodisk, the coexisting cavity and edge plasmon modes at a single wavelength results in either bright or dark spots on SNOM maps measured on gold nanodisks [68] and graphene nanoresonators with a disk and square shape [69].

To conclude, for the disk antenna geometry, we showed that near-field light of the SNOM aperture probe locally excites the higher order ${TE}_{mn}^{ij}$ CMs and WGMs in the *α*-Si nanodisk. Within our experimental spectral range, we observed modes with indices *m* = 1–5 and *n* = 1–3. The parity (*i*, *j*) of the excited modes can be either even or odd for two orthogonal symmetry mirror planes of the disk. The visualized TE_*mn*_ modes are different from the higher order TM_*mn*_ modes (*E*_*z*_ component is dominant, and *m* = 0–4, *n* = 1–4) that were observed by electron beam excitation of a Si-nanodisk [37] and an apertureless SNOM approach [52]. In our study, the simulations of near-field distributions show that the positions where the probe excites optical modes, coincide with |*H*_*z*_| nodes of the ${TE}_{mn}^{ij}$ modes. The SNOM maps do not directly represent the field distribution of individual optical modes, but rather a superposition due to the coexistence of a rich variety of modes. The excitation of disk modes results in either bright (high scattering) or dark (low scattering) spots on SNOM maps. The varying intensity contrast seen in the SNOM maps is associated with the scattering intensity of each excited mode, Fano-interference between the mode and probe radiation, and varying coupling efficiency of the probe’s near-field with the optical modes at their different nodes.

**Figure 2:**
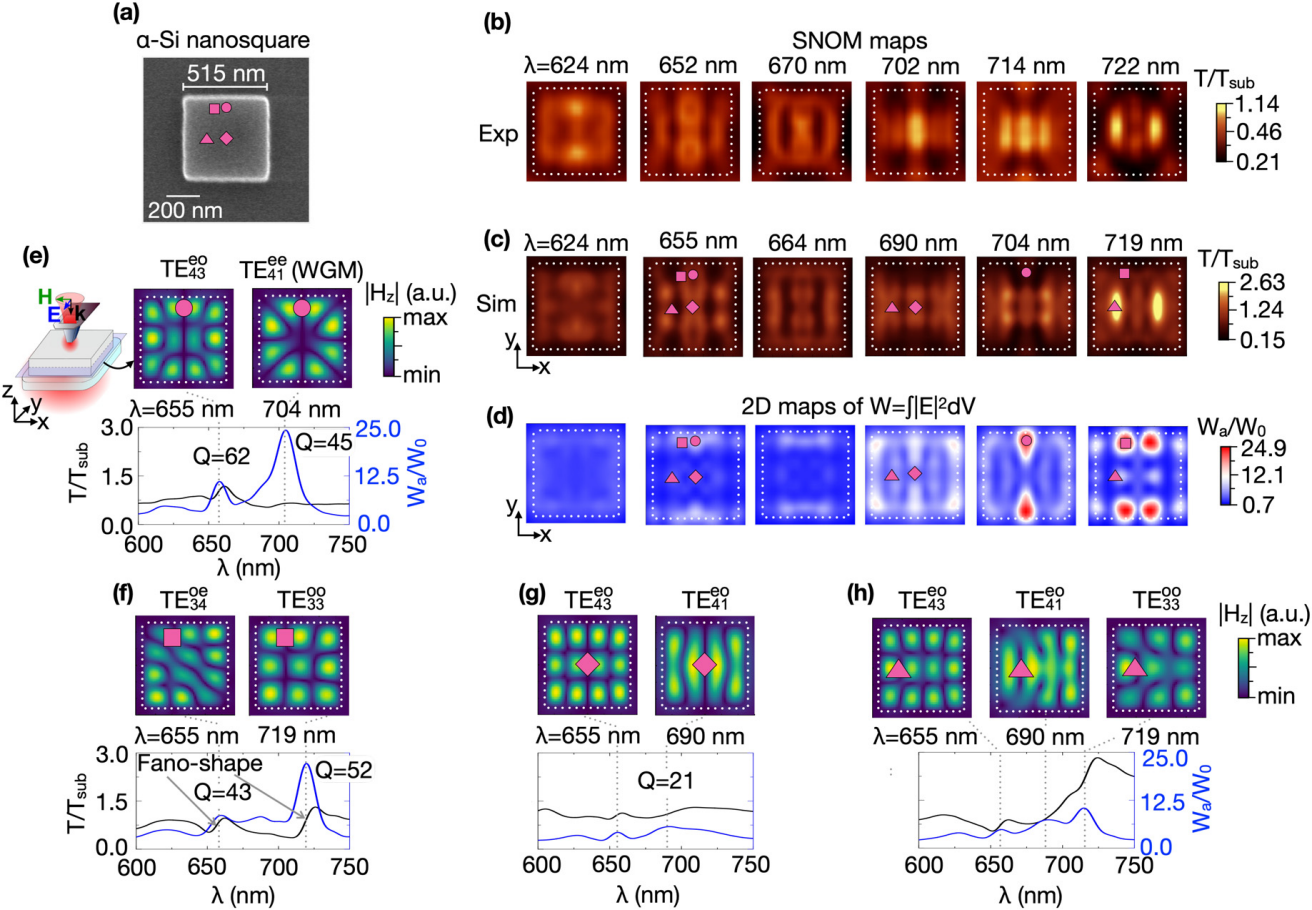
(a) SEM image of the *α*-Si nanosquare. The side length is 515 nm, and the thickness is 96 nm. Experimental (b) and simulated (c) SNOM maps of the *α*-Si nanosquare at different wavelengths. The polarization of the light incident on the SNOM probe (blue arrow) is directed along the *y*-axis. (d) Simulated 2D maps of electric field localization W_*a*_/W_0_ at the same wavelengths of the SNOM maps in (c). The circle (*x* = 0 nm, *y* = 220 nm), square (*x* = 80 nm, *y* = 200 nm), diamond (*x* = 0 nm, *y* = 0 nm), and triangle (*x* = 120 nm, *y* = 0 nm) symbols on 2D maps in (a), (c), and (d) depict the SNOM probe positions corresponding to maxima of electric field localization. (e–h) The simulated SNOM transmittance *T*/*T*_sub_ (black curves) and electric field localization W_*a*_/W_0_ (blue curves) spectra recorded at the fixed positions of the SNOM probe marked as purple symbols on SNOM (c) and W_*a*_/W_0_ (d) maps. The top rows in (e–h) show the simulated |*H*_*z*_| distributions of ${TE}_{mn}^{ij}$ optical modes. The |*H*_*z*_| field was taken at central *xy*-cross-sections of the nanosquare as shown in the sketch in panel (e). Dotted white lines indicate the boundary of the nanosquare.

### 2.2 Square

Next, we reduce the antenna rotational symmetry to four-fold and turn our attention to an *α*-Si nanosquare with dimensions similar to the nanodisk above: side length of 515 nm and thickness of 96 nm (see SEM image in Figure 2(a)). The experimental and simulated SNOM maps are shown in Figure 2(b) and (c), respectively, at multiple wavelengths between *λ* = 600 nm and *λ* = 750 nm. Also here, very distinct transmission patterns are obtained with a very good agreement between experiment and simulation. Although these patterns present some resemblance to those of the nanodisk, there are some clear differences. The calculated 2D maps of electric field localization W_*a*_/W_0_ shown in Figure 2(d) reveal the probe positions of resonant mode excitation at the local W_*a*_/W_0_ maxima. The prominent W_*a*_/W_0_ maxima positions – near and far from the square’s edges – are again marked in Figure 2(a) and (d) with a purple symbol. To find the exact wavelengths of resonant mode excitation, W_*a*_/W_0_ (blue curves) spectra taken at fixed probe positions are shown in Figure 2(e–h). The top row depicts the distribution of the dominant field component |*H*_*z*_| in the nanosquare taken at wavelengths corresponding to W_*a*_/W_0_ maxima in the plots at the bottom row. The |*H*_*z*_| distributions reveal that the probe excites ${TE}_{mn}^{ij}$ optical modes, which have |*H*_*z*_| as the dominant contribution to the total |**H**| field. The subscripts *m* and *n* in ${TE}_{mn}^{ij}$ indicate the number of |*H*_*z*_| antinodes in *x*, *y*-direction, respectively. There is only one antinode in the *z*-direction for the observed modes. Two W_*a*_/W_0_ maxima at *λ* = 655 nm and *λ* = 704 nm in panel (e) are associated with the excitation of ${TE}_{43}^{eo}$ CM and ${TE}_{41}^{ee}$ WGM (subscripts for ${TE}_{41}^{ee}$ mode correspond to the designation of a WGM in a disk) when the probe locates at the position near the square’s edge marked with the circle. At the square mark position, shown in panel (c) at *λ* = 655 nm, the SNOM probe excites the ${TE}_{43}^{eo}$ mode. The SNOM probe also launches the same ${TE}_{43}^{eo}$ mode at two other positions. The first is at the center of the nanosquare (panel (g), diamond, *λ* = 655 nm). The second is near the left edge (panel (h), triangle, *λ* = 655 nm). In addition to the excitation of the ${TE}_{43}^{eo}$ mode with even parity with respect to the *σ*_*x*_-plane and odd parity with respect to the *σ*_*y*_-plane, panel (f) shows the excitation of ${TE}_{34}^{oe}$ at the off-center probe position marked with the square (*λ* = 655 nm) having the opposite symmetry to ${TE}_{43}^{eo}$ . Along with ${TE}_{34}^{oe}$ , the probe excites ${TE}_{33}^{oo}$ at the square mark position and *λ* = 719 nm (Figure 2(f)). This mode is again launched at the triangle mark position at *λ* = 719 nm (panel (h)), but with smaller efficiency, since this position does not precisely correspond to the maximum of W_*a*_/W_0_ (see 2D W_*a*_/W_0_ map in panel (d)). The last CM observed is ${TE}_{41}^{eo}$ which is excited at *λ* = 690 nm at the diamond (panel (g)) and triangle (panel (h)) mark. As it was for the disk case, probe positions corresponding to resonant excitation coincide with |*H*_*z*_| nodes of these ${TE}_{mn}^{ij}$ modes.

Similar to the SNOM maps of the nanodisk, superimposing the W_*a*_/W_0_ maxima in Figure 2(d) onto the SNOM maps in Figure 2(c) highlights the transmittance features associated with the excitation of the optical modes. The excitation of multiple coexisting modes at their various |*H*_*z*_| nodes results in bright and dark spots on the SNOM maps. In panel (c), e.g., the bright and dark areas at *λ* = 655 nm are associated with the excitation of ${TE}_{43}^{eo}$ and ${TE}_{34}^{oe}$ CMs. ${TE}_{43}^{eo}$ excited at its central |*H*_*z*_| node (square mark) results in the local dark spot on the SNOM map, while excitation of ${TE}_{43}^{eo}$ at its off-center nodes results in a transmission intensity somewhere between the bright and dark spots (circle and triangle). Similar to the disk case, the excitation of ${TE}_{43}^{eo}$ CM at either its center or off-center |*H*_*z*_| node positions, gives rise to a different *T*/*T*_sub_ value, an effect that can be ascribed to a position dependent coupling efficiency between probe and mode. However, compared to the disk case, the *T*/*T*_sub_ maxima/minima show a larger spatial shift relative to the W_*a*_/W_0_ maxima as a result from the excitation of the square’s ${TE}_{43}^{eo}$ mode at its off-center nodes (circle and triangle). The intermediate *T*/*T*_sub_ values observed at W_*a*_/W_0_ maxima positions can intuitively be explained by the probe position dependence of the phase shift between the optical mode radiation and non-resonant probe radiation. When the probe is closer to/farther from the antenna center, the phase shift decreases/increases since the excited mode does not change its spatial position with respect to the antenna center during the scanning process. It results in the decrease/increase of the transmitted light (dark/bright areas) near the |*H*_*z*_| node positions. Suppose one of the two channels (antenna mode or non-resonant probe radiation) is negligible. In that case, the phase shift change does not lead to a significant spatial shift of the *T*/*T*_sub_ minima/maxima with respect to the W_*a*_/W_0_ maxima (as it was observed for the disk case). Following this, the phase change effect is more pronounced for ${TE}_{43}^{eo}$ modes than for the previously discussed ${TE}_{mn}^{ij}$ modes in the nanodisk due to the (approximately) equal contribution of the resonant and non-resonant radiation. Similar behavior is observed on the SNOM map at *λ* = 690 nm for the excitation of the ${TE}_{41}^{eo}$ mode at its various |*H*_*z*_| nodes. The local bright spot appears on the SNOM map at *λ* = 690 nm at the central |*H*_*z*_| node position of ${TE}_{41}^{eo}$ (diamond, panel (c)). However, the off-center node of this mode (triangle, panel (c)) is between the bright and dark areas. For the SNOM map at *λ* = 704 nm, the node of ${TE}_{41}^{ee}$ WGM is also between the bright and dark areas (panel (c), circle). For the SNOM map at *λ* = 719 nm, ${TE}_{33}^{oo}$ mode excitation results in two local bright spots with different relative *T*/*T*_sub_ at the square and triangle mark.

The different *T*/*T*_sub_ values, associated with excitation of optical modes, are also demonstrated in Figure 2(e–h) by the simulations of the *T*/*T*_sub_ (black curves) and W_*a*_/W_0_ (blue curves) spectral dependences recorded at individual SNOM probe positions marked with the purple dots. Similar to the disk case, *T*/*T*_sub_ curves present asymmetric Fano lineshapes near the vicinity of optical mode excitation (W_*a*_/W_0_ maxima) caused by interference of the antenna mode radiation and non-resonantly transmitted light. This again shows that the different modes result in various *T*/*T*_sub_ Fano lineshapes. Even for the individual modes, ${TE}_{43}^{eo}$ and ${TE}_{33}^{oo}$ , excited at their different |*H*_*z*_| nodes, the *T*/*T*_sub_ values and Fano lineshapes differ. The different Fano lineshapes are also associated with changes in a phase shift and coupling efficiency when modes are excited at their different |*H*_*z*_| nodes.

To summarize for the square geometry, SNOM maps of the *α*-Si nanosquare reveal the excitation of higher order ${TE}_{mn}^{ij}$ WGM ((*m*, *n*) = (4, 1)) and CMs ((*m*, *n*) = (4, 1); (3, 3); (3, 4); (4, 3)) with either even or odd parity (*i*, *j*). The observed chess-like |*H*_*z*_| field distribution of ${TE}_{mn}^{ij}$ CMs in the square antenna differs from the disk case due to the difference in the light reflection conditions at the two opposite parallel edges of the square compared to the rounded edges in the disk antenna cavity. Similar to the disk case, the intensity contrast of each SNOM map at a single wavelength results from the superposition of spectrally overlapping higher order ${TE}_{mn}^{ij}$ CMs and WGMs. The SNOM probe positions of the resonant excitation occurring at |*H*_*z*_| nodes of ${TE}_{mn}^{ij}$ modes, coincide with local bright and dark areas on the SNOM maps as was for the disk case, but now also at intermediate transmission values. The SNOM contrast is again associated with various scattering intensities of the excited modes, the Fano-interference between the probe and mode radiation, and the different coupling efficiency of the probe near-field to the modes.

### 2.3 Triangle

**Figure 3:**
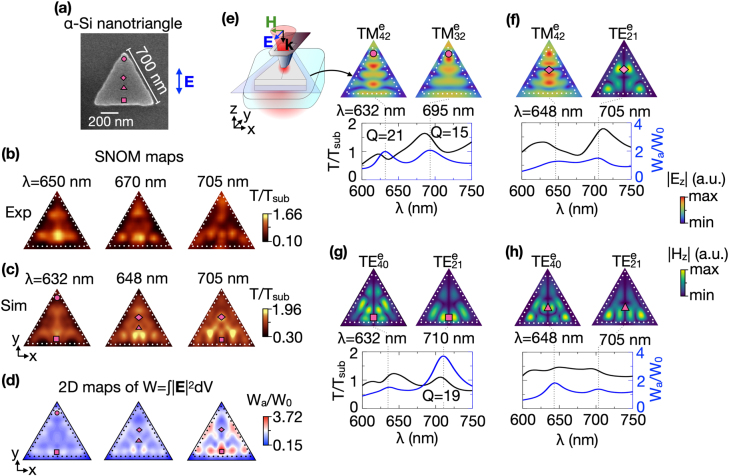
(a) SEM image of the equilateral *α*-Si nanotriangle. The side length of triangle is 700 nm, and the thickness is 100 nm. The blue arrow indicates the *y*-polarization of light focused in the SNOM probe. Experimental (b) and simulated (c) SNOM maps of the *α*-Si equilaterale nanotriangle for *y*-polarized excitation. (d) Simulated 2D maps of electric field localization W_*a*_/W_0_ at the same wavelengths of the SNOM maps in (c). The circle (*x* = 0 nm, *y* = 580 nm), square (*x* = 0 nm, *y* = 120 nm), diamond (*x* = 0 nm, *y* = 360 nm), and triangle (*x* = 0 nm, *y* = 260 nm) symbols on (a), (c), and (d) depict the SNOM probe positions corresponding to maxima of electric field localization. (e–h) The simulated SNOM transmittance *T*/*T*_sub_ (black curves) and electric field localization W_*a*_/W_0_ (blue curves) spectra recorded at the fixed positions of the SNOM probe marked as purple symbols on the SNOM (c) and W_*a*_/W_0_ (d) maps. The top rows in (e–h) show the simulated |*E*_*z*_| and |*H*_*z*_| distributions for ${TM}_{mn}^{e}$ and ${TE}_{mn}^{e}$ modes, respectively. The |*E*_*z*_| and |*H*_*z*_| field was taken at central *xy*-cross-sections of the nanotriangle as shown in the sketch in panel (e). Dotted white and black lines indicate the boundary of the nanotriangle.

**Figure 4:**
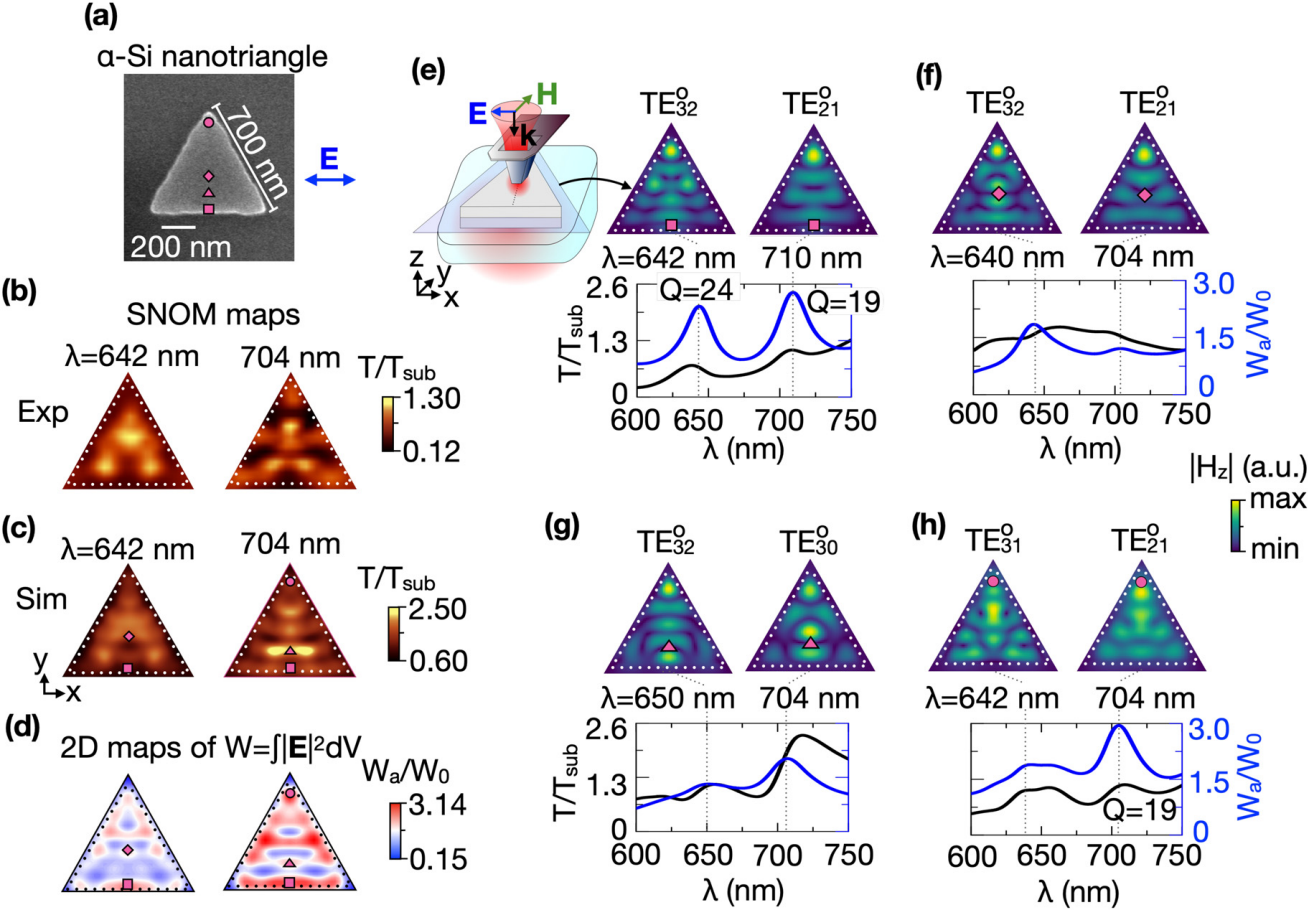
(a) SEM image of the equilateral *α*-Si nanotriangle (the same as in Figure 3). The side length of triangle is 700 nm, and the thickness is 100 nm. The blue arrow indicates the *x*-polarization of light focused in the SNOM probe. Experimental (b) and simulated (c) SNOM maps of the *α*-Si equilaterale nanotriangle for *x*-polarized excitation, respectively. The blue arrow indicates the *x*-polarization of light focused in the SNOM probe. (d) Simulated 2D maps of electric field localization W_*a*_/W_0_ at the same wavelengths of the SNOM maps in (c). The circle (*x* = 0 nm, *y* = 560 nm), square (*x* = 0 nm, *y* = 80 nm), diamond (*x* = 0 nm, *y* = 260 nm), and triangle (*x* = 0 nm, *y* = 180 nm) symbols on (a), (c), and (d) depict the SNOM probe positions corresponding to maxima of electric field localization. (e–h) The simulated SNOM transmittance *T*/*T*_sub_ (black curves) and electric field localization W_*a*_/W_0_ (blue curves) spectra recorded at the fixed positions of the SNOM probe marked as purple symbols on SNOM (c) and W_*a*_/W_0_ (d) maps. The top rows in (e–h) show the simulated |*H*_*z*_| distributions of ${TE}_{mn}^{o}$ modes. The |*H*_*z*_| field was taken at central *xy*-cross-sections of the nanotriangle as shown in the sketch in panel (e). Dotted white and black lines indicate the boundary of the nanotriangle.

Reducing the rotational symmetry of the nanoantenna further expands the modal spectra. In contrast to the disk and square shape, for the equilateral triangle possessing mirror symmetry only with respect to the *σ*_*x*_-plane, a 90° change in the excitation polarization allows the SNOM probe to couple to a different set of modes [70]. Therefore, the polarization directions parallel and perpendicular with respect to the triangle’s height (*y*-axis) are studied separately in Figures 3 and 4, respectively. Starting with parallel polarization in Figure 3, the experimental SNOM maps of an equilateral *α*-Si nanotriangle with a side length of 700 nm and thickness of 100 nm (see SEM image in panel (a)) are shown in panel (b) together with the simulated results in panel (c). Again, the simulated SNOM maps demonstrate a good agreement with the experimental results. Maxima on the 2D W_*a*_/W_0_ maps in Figure 3(d) marked with the purple symbols indicate probe positions that result in resonant mode excitation. For the triangle antenna, we limit our discussion to probe positions on the triangle’s height from the top corner to the base.

The field distribution in Figure 3(e–h) taken at the fixed probe positions (marked by purple symbols in panel (d)) and corresponding to maxima of W_*a*_/W_0_ spectra (blue curves) in Figure 3(e–h) demonstrate the excitation of both ${TM}_{mn}^{i}$ and ${TE}_{mn}^{i}$ modes with dominant |*E*_*z*_| and |*H*_*z*_| components, respectively. The ${TM}_{mn}^{i}$ as well as the ${TE}_{mn}^{i}$ modes can be excited by the SNOM probe due to the existence of two intense |*E*_*z*_| hotspots near the SNOM probe aperture along the *y*-direction. These two |*E*_*z*_| hotspots resonantly couples with two |*E*_*z*_| antinodes of ${TM}_{mn}^{i}$ modes at their |*E*_*z*_| node positions. More details on the distributions of all field components near the aperture SNOM probe can be found in the Supporting Information of Ref. 56. The indices *m* and *n* refer to analytical solutions for TM and TE waveguide modes with even and odd parity (with respect to the *σ*_*x*_-plane perpendicular to the *x*-axis) for a hollow equilateral triangle waveguide with metal walls [71, 72]. These modes have a similar spatial arrangement of antinodes as the dielectric triangle cavity [70, 73] (see analytical solutions in Section 4, Supplementary Materials). The *E*_*z*_ component is transformed under mirror reflection with respect to the *σ*_*x*_-plane with symmetric function (*E*_*z*_(−*x*, *y*) = *E*_*z*_(*x*, *y*)) for even symmetry modes, while the *H*_*z*_ component is transformed as an antisymmetric function (*H*_*z*_(*x*, *y*) = −*H*_*z*_(−*x*, *y*)) since **H** is a pseudovector. When the probe locates near the top corner (panel (e), circle), ${TM}_{42}^{e}$ and ${TM}_{32}^{e}$ modes with the dominant |*E*_*z*_| component are excited at *λ* = 632 nm and *λ* = 695 nm, respectively, having even symmetry with respect to the *σ*_*x*_-plane. ${TM}_{42}^{e}$ is also excited at the position marked with the diamond in Figure 3(f) at *λ* = 648 nm. At this position, the probe also excites ${TE}_{21}^{e}$ at *λ* = 705 nm (Figure 3(f)). Figure 3(g) shows excitation of ${TE}_{40}^{e}$ (*λ* = 632 nm) and ${TE}_{21}^{e}\;\lambda =710$ nm) when the probe locates near the bottom side of the triangle (position marked with the square). Figure 3(h) demonstrates that ${TE}_{40}^{e}$ (*λ* = 632 nm) and ${TE}_{21}^{e}$ (*λ* = 705 nm) modes are excited again at the position marked with the triangle.

The positions of resonant excitation coincide with |*E*_*z*_| nodes for ${TM}_{mn}^{e}$ and |*H*_*z*_| nodes for ${TE}_{mn}^{e}$ . These positions, superimposed on the SNOM maps (purple symbols in Figure 3(c)), show that coexisting optical modes result in various *T*/*T*_sub_ contrast on an SNOM map due to their different scattering intensities and Fano-interference between the non-resonant probe and antenna radiation. For the SNOM map at *λ* = 632 nm (Figure 3(c)), the local dark spot near the triangle’s corner (see circle at *λ* = 632 nm in Figure 3(c)) results from ${TM}_{42}^{e}$ excitation. While the square mark position near the triangle’s bottom (panel (c)), corresponding to ${TE}_{40}^{e}$ excitation, is located between a bright and dark area. For the SNOM map at *λ* = 648 nm, the local bright spot at the triangle mark position and the dark one at the diamond mark position appear due to the ${TE}_{40}^{e}$ and ${TM}_{42}^{e}$ modes excitation, respectively. Finally, for the SNOM map at *λ* = 705 nm, the first |*H*_*z*_| node of ${TE}_{21}^{e}$ coincides with the local bright spot at the diamond mark, while the second one locates between bright and dark areas (square). Such difference of *T*/*T*_sub_ value for the ${TE}_{21}^{e}$ mode is again associated with the different coupling efficiency of the probe near-field with the ${TE}_{21}^{e}$ mode at two different |*H*_*z*_| node positions. The second reason is a different phase shift between the resonant and non-resonant radiation for the two different probe positions.

Figures 4(b) and (c) show that rotation of the excitation polarization by 90° leads to modification of the SNOM maps due to the absence of mirror symmetry of the nanotriangle with respect to the *σ*_*y*_-plane (perpendicular to the *y*-axis). Again, experimental maps (panel (b)) are in very good agreement with simulated results (panel (c)). The selected wavelengths of *λ* = 642 nm and *λ* = 704 nm correspond to the maxima on the W_*a*_/W_0_ maps shown in panel (d). The simulated |*H*_*z*_| distributions shown in panels (e–h) demonstrate that the type of modes excited along the height of the triangle is transverse electric with odd parity with respect to the *σ*_*x*_-plane. The mode parity is opposite to the one observed for *y*-polarized illumination (Figure 3). The *H*_*z*_ field distribution of the modes for *x*-polarized illumination is transformed as a symmetric function (*H*_*z*_(*x*, *y*) = *H*_*z*_(−*x*, *y*)). The ${TE}_{32}^{o}$ (*λ* = 642 nm) and ${TE}_{21}^{o}$ (*λ* = 710 nm) modes (panel (e)) are excited at the position marked with the square near the bottom side of the triangle. ${TE}_{32}^{o}$ and ${TE}_{21}^{o}$ are also excited when the probe locates near the center of the nanotriangle (diamond in panel (f)). ${TE}_{21}^{o}$ is also excited near the corner of the nanotriangle (panel (h), circle at *λ* = 704 nm). Panel (g) shows the distribution of a ${TE}_{30}^{o}$ mode at *λ* = 704 nm which is excited at the triangle mark probe position. One more mode is ${TE}_{31}^{o}$ which is excited near the top corner (circle in panel (h) at *λ* = 642 nm).

The superimposed W_*a*_/W_0_ maxima positions on the simulated SNOM maps (Figure 4(c)) show the various SNOM probe transmittance features associated with the excitation of coexisting optical modes with different scattering intensity, similar to the disk and square case. For the SNOM map at *λ* = 642 nm, ${TE}_{32}^{o}$ excitation results in the local dark spot at the triangle’s bottom (square mark) and the bright spot at the diamond mark position. At *λ* = 704 nm, the local bright and dark spot near the triangle’s bottom (square mark) and corner (circle mark), respectively, result from ${TE}_{21}^{o}$ mode excitation. Another local bright spot at the triangular mark position corresponds to ${TE}_{30}^{o}$ mode excitation. As is the case for the disk and square geometry, Fano resonance lineshapes in the transmission spectra, recorded at fixed probe positions, are also observed for the nanotriangle’s ${TE}_{nm}^{i}$ and ${TM}_{nm}^{i}$ modes, and this for both excitation polarizations (see *T*/*T*_sub_ spectra in panels (e–h) in Figures 3 and 4).

Concluding the triangle geometry, the bright and dark spots on the observed SNOM maps are associated with excitation of ${TE}_{mn}^{i}\;\left(\left(m,n\right)=\left(4,0\right),\left(3,2\right),\left(3,1\right),\left(3,0\right),\left(2,1\right)\right)$ and ${TM}_{mn}^{i}\;\left(\left(m,n\right)=\left(4,2\right),\left(3,2\right)\right)$ higher order optical modes. The dark and bright features are linked with ${TE}_{mn}^{i}$ or ${TM}_{mn}^{i}$ modes by simulations of the electric field localization and field distributions. Similar to the disk and square antennas, the bright and dark features on the SNOM maps appear due to the differences in scattering intensities of the excited modes, the Fano-interference between the probe and mode radiation, and difference in coupling efficiency of the probe near-field with the modes. In addition, the change of the illumination polarization leads to a modification of the mode parity of the excited optical modes. The positions of resonant excitation coincide with |*E*_*z*_| nodes for ${TM}_{mn}^{e}$ , and |*H*_*z*_| nodes for ${TE}_{mn}^{e}$ and ${TE}_{mn}^{o}$ .

### 2.4 Quality factor and light trapping capabilities of the excited modes

In addition to the far-field radiative properties of the locally excited higher order modes, we estimate their *Q*-factors and light trapping capabilities, which are important parameters for applications in light-emitting devices, solar cells, and non-linear light generation. For this, the W_*a*_/W_0_ spectra of the nanodisk (Figure 1(f) and (g)), nanosquare (Figure 2(e)–(g)), and nanotriangle (Figure 3 (e and g) and Figure 4(e and h)) were fitted with a multi-peak Lorentzian. For the nanodisk, the highest quality factor of *Q* = 68 and highest field localization value of W_*a*_/W_0_ = 7.5 are obtained for ${TE}_{41}^{ee}$ WGM. These values are higher in comparison with other higher order CMs of the nanodisk (*Q* = 24 and W_*a*_/W_0_ = 2.3 for ${TE}_{13}^{eo}$ ) and the lowest order modes in a Si nanodisk (magnetic or electric dipole modes [74]).

A slightly lower *Q* = 45 is observed for ${TE}_{41}^{ee}$ WGM (Figure 2(e)) in the nanosquare. This can be attributed to the additional scattering experienced by the WGM at the sharp corners of the square. The cavity modes in the nanosquare, on the other hand, have *Q* = 63 and *Q* = 52 for ${TE}_{43}^{eo}$ (Figure 2(e)) and ${TE}_{33}^{oo}$ (Figure 2(f)), respectively. This is higher in comparison to the CMs in the nanodisk and comparable with its ${TE}_{41}^{ee}$ WGM. A higher *Q*-factor for the CMs in the nanosquare can be explained by its well-defined cavity length defined by the two opposite parallel edges, which are absent in a disk and triangle. A lower *Q*-factor of 21 is observed for the ${TE}_{41}^{eo}$ CM in the nanosquare. Interestingly, for the ${TE}_{33}^{oo}$ CM and ${TE}_{41}^{ee}$ WGM in the nanosquare, very strong field localization reaching almost W_*a*_/W_0_ = 25 is observed. This means that these modes can efficiently trap light within the nanoantenna due to the high coupling efficiency to the probe near-field.

The estimated *Q*-factors of the nanotriangle modes are generally lower than the maximum values observed for the nanosquare antenna and are compared to the nanodisk CMs. For *y*-polarized illumination, we obtained *Q*-factors of *Q* = 21 for ${TM}_{42}^{e}$ , *Q* = 19 for ${TE}_{21}^{e}$ , and *Q* = 15 for ${TM}_{32}^{e}$ (Figure 3(e) and (g)). For *x*-polarized illumination we observed *Q*-factors of *Q* = 24 for ${TE}_{32}^{o}$ and *Q* = 19 for ${TE}_{21}^{o}$ (Figure 4(e)).

However, the SNOM probe–antenna interactions can modify the modal excitation boundary conditions as well as the absorption and scattering of the optical modes. To reveal the influence of the SNOM probe on the Q-factor and resonant wavelength, we compared these parameters calculated from the SNOM data (spectral dependence of electric field localization W_*a*_/W_0_) with eigenmode analysis without the SNOM probe. Tables S1–S3 in Supplementary Materials, Section 5 show results for optical modes in the nanodisk, nanosquare, and nanotriangle, respectively. For optical modes with Q > 40 (obtained from SNOM W_*a*_/W_0_ data), Q-factors obtained from eigenmode analysis are almost two times larger than the results from SNOM spectral data. In contrast, the eigenmode analysis has similar values for the low Q optical modes (Q < 30 obtained from the SNOM data analysis). The more significant difference of the Q-factors for high Q modes (Q > 40) can be associated with the influence of the SNOM probe on excited optical modes. The SNOM probe leads to the additional light scattering and absorption of the optical modes that decrease their Q-factor. Low Q modes already have multiple channels for radiative and absorption losses. Therefore, adding one more channel (an SNOM probe) will not substantially impact the new Q-factor. In contrast, the impact on a high Q mode will be more prominent. Tables S1–S3 also show that the SNOM probe has only a small impact on the resonant wavelengths. This can be explained by the localization of the optical modes inside the volume of the nanoantennas.

It should be noted that the excitation of the optical modes near nanoantennas’ edges leads to higher electric field localization than at other probe positions. ${TE}_{43}^{eo}$ excited near the edge of the nanosquare leads to W_*a*_/W_0_ = 11 (Figure 2(e), circle mark) in comparison to 4.3 and 4.7 at two other positions (diamond mark in Figure 2(g) and triangle mark in Figure 2(h)). Also, for the nanosquare, excitation of ${TE}_{33}^{oo}$ near the square edge leads to almost W_*a*_/W_0_ = 25 (Figure 2(f)), which is considerably higher than W_*a*_/W_0_ = 12 obtained at the position near the square center (Figure 2(h)). The excitation of ${TE}_{21}^{e}$ (Figure 3(g)), ${TE}_{32}^{o}$ (Figure 4(e)), and ${TE}_{21}^{o}$ (Figure 4(h)) in the nanotriangle results in higher field localization (W_*a*_/W_0_ is around 3) at the triangle’s corner and base than other excitation positions.

The variable coupling efficiency that is observed originates from differences in the field overlap of the optical modes and near-field around the SNOM probe. This overlap is strongly governed by the spatial distribution of the field antinodes. Depending on the mode spatial structures, the field antinodes can be closer or farther from each other. It also depends on the wavelength of the excitation. For the disk, square, and triangle nanoantennas, the spatial distribution of the excited optical modes is different, which modifies the overlap of the optical modes with the near-field of the SNOM probe. The coupling efficiency is also governed by their intrinsic losses. The ratio of the radiative and absorption loss of the optical modes determines the coupling efficiency [75, 76]. All incident light can be coupled with the optical antenna modes at the equivalence between these two quantities. In other cases, the SNOM probe near-field light is more efficiently absorbed or scattered. As each mode has its own radiative loss, different modes possess a different coupling efficiency with the SNOM probe near-field.

### 2.5 Plane wave excitation

The higher order CMs and WGMs studied above by the SNOM probe excitation are difficult to be observed by far-field techniques using a plane wave source. For comparison, in Section 6, Supplementary Materials, we applied a plane wave source with normal incidence to drive the optical modes in the nanoantenna. It is shown that for the nanodisk, nanosquare, and nanotriangle such an approach allows the coupling to only a limited number of modes. The inability to excite all antenna supported modes is associated with a symmetry mismatch of the electromagnetic fields of the modes and the plane wave source. Moreover, even for the symmetry-allowed modes studied above, low coupling efficiency with a normal-incidence plane wave source complicates their excitation and observation.

## 3 Conclusions

Aperture type scanning near-field optical microscopy (SNOM) reveals a very rich variety of near-field optical patterns in amorphous silicon nanoantennas. The subwavelength localized excitation of higher order optical cavity modes (CMs) and whispering gallery modes (WGMs) strongly depends on the nanoantennas shape, probe position, and excitation frequency. This has been demonstrated for some of the most basic geometries with varying rotational symmetries: a disk, square, and triangle. A detailed analysis based on full-field FDTD simulations allowed us to link the aperture SNOM maps to the field distributions of higher order modes supported by the nanoantennas which have not been previously visualized. The experimental data has been reconstructed based on the decomposition in CMs and WGMs with different order and parity. The insights obtained within this study for basic antenna cavity shapes provide an important foundation for the detailed understanding of the optical behavior of more complex nanocavity systems under local excitation and the interpretation of experimental near-field data. The local excitation of CMs and WGMs with even and odd symmetry is accompanied by very distinct light scattering and trapping properties that have a direct impact on e.g. the quantum efficiency of nearby emitters, the efficiency of non-linear effects [77, 78], and directionality of scattered or emitted light [19, 79].

## Supplementary Material

Supplementary Material Details
