# Supplementary material for: Probing higher order optical modes in all-dielectric nanodisk, -square, and -triangle by aperture type scanning near-field optical microscopy
Source: Nanophotonics. 2021 Dec 22;11(3):543–57. doi: 10.1515/nanoph-2021-0612 (PMC11501575; doi:10.1515/nanoph-2021-0612)
Supplement: Supplementary file 1 — Supplementary Material Details [file j_nanoph-2021-0612_suppl.pdf]

Supplementary Materials

Aleksandr Yu. Frolov*, Joris Van de Vondel, Vladimir I. Panov, Pol Van Dorpe, Andrey A. Fedyanin, Victor V. Moshchalkov, and Niels Verellen

Supplementary Materials for "Probing higher order optical modes in all-dielectric nanodisk, –square, and –triangle by aperture type scanning near-field optical microscopy"

1 Methods

1.1 Sample fabrication

Amorphous silicon (α-Si) thin films were deposited using radio frequency plasma-enhanced chemical vapor deposition (RF-PECVD) on a glass substrate with refractive index of $n = 1.52$. Nanodisks, nanosquares and nanotriangles were subsequently patterned by electron beam lithography and inductively coupled plasma (ICP) SF_6/CF_4 dry etching according to our previously reported recipe[1]. To exclude near- and far-field coupling between nanoantennas in the array, a pitch of 4 μm was used. The thickness of the nanoantennas was characterized by atomic force microscopy (AFM) (Supporting Information Figure S1), and the lateral dimension was determined by scanning electron microscopy. The dimensions are nanodisk diameter$\times$thickness= 515 nm $\times$ 96 nm, nanosquare length$\times$thickness= 515 nm $\times$ 96 nm, equilateral nanotriangle length$\times$ thickness= 700 nm $\times$ 100 nm. To accurately reproduce the spectral positions of the excited optical modes, the thickness of the disk and square were slightly adapted in the FDTD simulations to 90 nm for the nanodisk and nanosquare. The $\alpha-$Si high refractive index $n = 3.66 - 4.0$ and low extinction coefficient $k < 0.085$ in the visible and near-infrared range from 600 nm to 750 nm provide a high concentration of the electromagnetic field with low absorption losses[1].

***Corresponding author: Aleksandr Yu. Frolov,** Lomonosov Moscow State University, Faculty of Physics, Moscow, Russian Federation e-mail: frolov@nanolab.phys.msu.ru
Joris Van de Vondel, KU Leuven, Department of Physics and Astronomy, Quantum Solid-State Physics Leuven, Belgium, e-mail: joris.vandevondel@kuleuven.be
Vladimir I. Panov, Lomonosov Moscow State University, Faculty of Physics, Moscow, Russian Federation e-mail:panov@spmlab.phys.msu.ru
Pol Van Dorpe, imec, Leuven, Belgium; Department of Physics and Astronomy, Quantum Solid-State Physics, KU Leuven, Leuven, Belgium, e-mail: Pol.VanDorpe@imec.be
Andrey A. Fedyanin, Lomonosov Moscow State University, Faculty of Physics, Moscow, Russian Federation e-mail:fedyanin@nanolab.phys.msu.ru
Victor V. Moshchalkov, Department of Physics and Astronomy, Quantum Solid-State Physics, KU Leuven, Leuven, Belgium, e-mail: victor.moshchalkov@kuleuven.be
Niels Verellen, imec, Leuven, Belgium; KU Leuven, Department of Physics and Astronomy, Quantum Solid-State Physics, KU Leuven, Leuven, Belgium, e-mail: Niels.Verellen@imec.be

1.2 Measurements and simulation

1. SNOM measurements. The near-field mapping of nanoantennas was performed with an aperture type scanning near-field optical microscope (WITec, alpha 300s[2]) in transmission illumination mode. The SNOM probe consists of a hollow SiO_2 pyramid coated with a 100 nm Al layer. A hole of 100 nm in diameter is etched at the pyramid's apex. This subwavelength hole provides the optical resolution below the diffraction limit. A supercontinuum white light laser (NKT Photonics, Koheras SuperK Extreme Standard) with an acousto-optical tunable filter (AOTF) was used as the light source. The AOTF selects simultaneously up to eight wavelength channels from the spectrum of the supercontinuum. The spectral width of each channel is around $10 - 20$ nm. After the passing through a polarizer, the light is focused on the apex of the probe by an objective with $20\times$ magnification, $NA = 0.4$. The light transmitted through the aperture probe and the nanoantenna was collected in the far-field by a reflective objective with $NA = 0.8$ and delivered to a spectrometer (Princeton Instruments) equipped with a Si CCD camera. The sample scanning was performed in atomic force microscopy contact mode with a spatial resolution of 20 nm.
2. SNOM simulations. The simulation of the scanning process was performed with a FDTD solver (Lumerical Solutions[3]). The probe was modeled as a hollow SiO_2 pyramid coated with a 100 nm thick Al layer. The diameter of the hole at the apex of the pyramid equals 90 nm. A broadband plane wave source ($\lambda = 600-750$ nm) is positioned inside the pyramid for optical mode excitation. The source's linear polarization was oriented either in the horizontal ($x-$axis) or vertical ($y-$axis) direction. The nanoantenna was moved with steps of 20 nm along the $x-$ and $y-$directions with respect to the SNOM probe. The mesh size in simulations was set to 10 nm in the $xy-$plane and 5 nm along the $z-$direction. The transmitted light intensity was recorded at each probe's position by the far-field projection monitor placed 20 nm below the bottom of the nanoantenna. The far-field projection provides the angular scattering distribution (i.e., radiation pattern), taking into account the refractive index change at the glass/air interface. To better approximate the experimental conditions, the NA of the collection objective was taken into account by calculating the transmission intensity through the same collection angle ($2\theta = 128°$). The SNOM transmitted intensity T was normalized to the one T_{sub} obtained without nanoantenna. To calculate the electric field localization W_a in the nanoantennas excited by the probe, an array of 2D monitors is placed inside the volume of the nanoantenna with a pitch of 5 nm along the $x-$axis. The electric field localization W_a is calculated as $W_a = \sum w_i \Delta V_i$, where $w_i = \int |E|^2 dS$ is the integral over each 2D monitor and ΔV_i is the volume between i and $i+1$ monitor. W_0 was calculated without the presence of the nanoantenna to normalize W_a.

2 Atomic force microscopy images

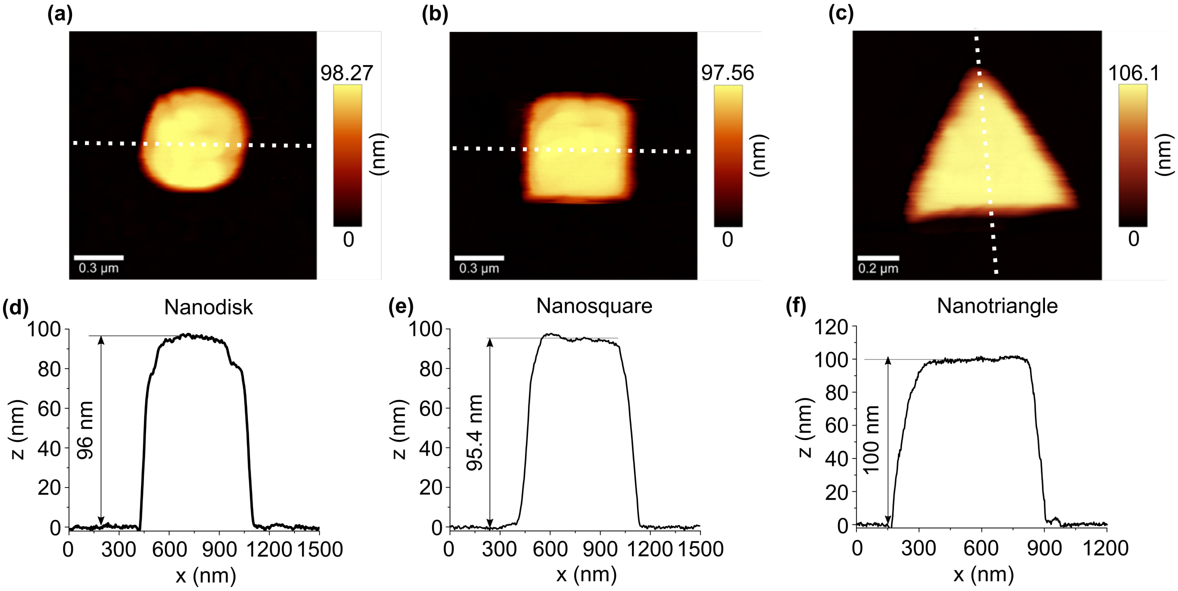

Fig. S1: Atomic force microscopy images of the α-Si nanodisk (a), – square (b), – triangle (c). (d,e,f) Cross-sections of the AFM images along the dotted lines in (a-c). From these scans we determine the antenna thickness to be 96 nm for the disk and square, and 100 nm for the triangle.

3 Simultaneous excitation of TE_{mn}^{ij} optical modes

An effect that complicates the analysis and direct interpretation of the SNOM maps is the excitation by the probe of mode superposition. This can occur when the individual modal antinodes and spectral bands overlap. As shown in the main text in Figure 1(g,h,i), the $|H_z|$ distribution of optical modes excited at square, diamond, and triangle mark positions demonstrate the complex superposition of disk cavity eigenmodes. We simulated the $|H_z|$ distribution of the individual eigenmodes of the α-Si infinite waveguide to show this clearly. Figure S2(a) demonstrates that the multiple $|H_z|$ antinodes, resulting from the SNOM probe excitation at $\lambda = 670$ nm, can be decomposed on the individual TE_{41}^{oo} WGM and TE_{13}^{eo} CM. Firstly, the simultaneous excitation of two modes takes place due to overlapping their spectral bands. The spectral positions of TE_{41}^{ee} WGM ($\lambda = 670$ nm) and TE_{13}^{eo} CM ($\lambda = 685$ nm) are close and, due to their large bandwidths, their spectral bands overlap (see W_a/W_0 spectra in Figure 1(f,g) in the main text). TE_{41}^{oo} WGM, which is degenerate with TE_{41}^{ee} WGM, supported by the nanodisk, is, therefore, also spectrally close to the TE_{13}^{eo} CM. Secondly, there are areas where the H_z field with the same phase sign is spatially overlapped for two modes. The areas of the spatial overlapping are marked as open circles in Figure S2(a).

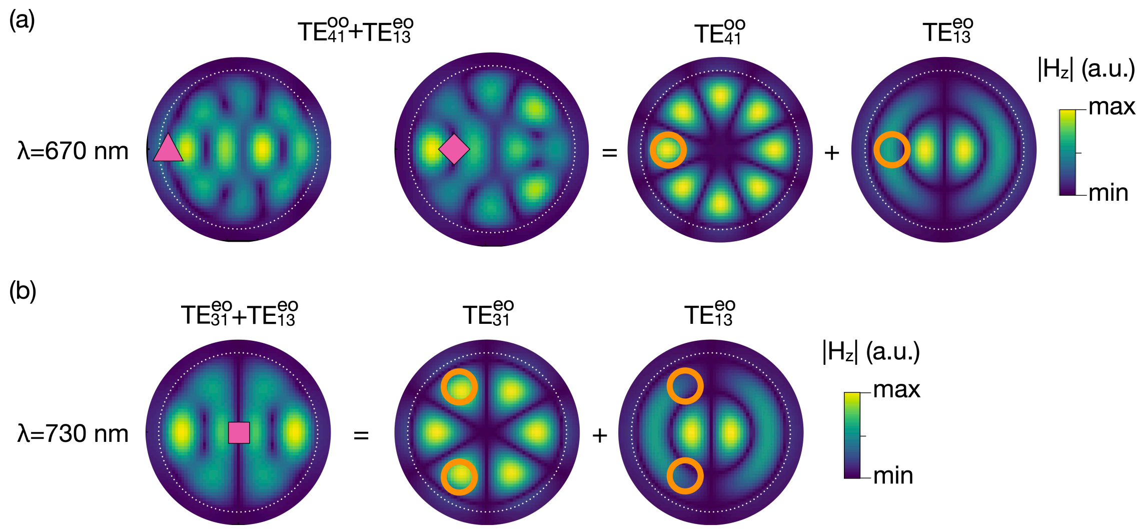

Fig. S2: (a) The decomposition of $|H_z|$ distribution of TE$^{ij}_{mn}$ modes excited by the SNOM probe in the α-Si nanodisk on eigenmodes of the α-Si infinite circular waveguide. (a) $\lambda = 670$ nm (b) $\lambda = 730$ nm. The purple symbols depict the SNOM probe positions. The open circle dots show areas where the H_z field with the same phase sign is spatially overlapped for two modes. The dotted circles depict the boundary of the disk and waveguide.

When now exciting with a single wavelength of $\lambda = 670$ nm at the triangle and diamond mark positions, both modes can couple to the probe resulting in a mixed TE$^{oo}_{41}$+TE$^{eo}_{13}$ mode. Analogously, Figure S2(b) demonstrates the decomposition of the H_z field distribution observed at square mark position ($\lambda = 730$ nm) on the TE$^{eo}_{31}$ WGM and TE$^{eo}_{13}$ CM.

4 Analytical solutions of modes in the hollow equilateral triangle with metal walls

For the designation of the modes excited in the nanotriangle, we used the analytical solutions of the wave equation in the hollow equilateral triangle with metal walls taken from Ref. 4, 5. The side length of the triangle equals 1. The σ_x is the symmetry mirror plane for the triangle geometry.

a) For TE$^{e}_{mn}$, the H_z magnetic field is proportional to the function:

$$\phi^{e}_{mn}(x,y) = \sin\left[\frac{(m+2n)\,\pi x}{3\sqrt{3}}\right]\cos\left[\frac{m\pi\,(2-y)}{3}\right] + \sin\left[\frac{(m-n)\,\pi x}{3\sqrt{3}}\right]\cos\left[\frac{(m+n)\,\pi\,(2-y)}{3}\right] - \sin\left[\frac{(2m+n)\,\pi x}{3\sqrt{3}}\right]\cos\left[\frac{n\pi\,(2-y)}{3}\right] \quad (1)$$

b) For TE^{o}_{mn}, the H_z magnetic field is proportional to the function:

$$\phi^{o}_{mn}(x,y) = \cos\left[\frac{(m+2n)\pi x}{3\sqrt{3}}\right]\cos\left[\frac{m\pi(2-y)}{3}\right] + \cos\left[\frac{(m-n)\pi x}{3\sqrt{3}}\right]\cos\left[\frac{(m+n)\pi(2-y)}{3}\right] + \cos\left[\frac{(2m+n)\pi x}{3\sqrt{3}}\right]\cos\left[\frac{n\pi(2-y)}{3}\right] \quad (2)$$

c) For TM^{e}_{mn}, the E_z electric field is proportional to the function:

$$\psi^{o}_{mn}(x,y) = \cos\left[\frac{(m+2n)\pi x}{3\sqrt{3}}\right]\sin\left[\frac{m\pi(2-y)}{3}\right] - \cos\left[\frac{(m-n)\pi x}{3\sqrt{3}}\right]\sin\left[\frac{(m+n)\pi(2-y)}{3}\right] + \cos\left[\frac{(2m+n)\pi x}{3\sqrt{3}}\right]\sin\left[\frac{n\pi(2-y)}{3}\right] \quad (3)$$

5 Comparison of the Q-factor and spectral wavelength calculated by eigenmode analysis and SNOM electric field localization data

An eigenmode analysis was performed by Lumerical FDTD Solver by using the quality factor analysis group [3]. It was based on the positioning of the electric dipole source at the field nodes of each optical mode (purple symbol positions in Figures 1-4 in the main text) and the Fourier transform of the decaying field of excited optical modes. Tables S1-S3 show the summarized comparison of the calculated Q-factor and resonant wavelength extracted from the eigenmode analysis and SNOM $\mathrm{W}_a/\mathrm{W}_0$ data for optical modes in α-Si nanodisk, -square, -triangle, respectively.

Tab. S1: Comparison of Q-factor and resonant wavelength of optical modes calculated by eigenmode analysis and SNOM $\mathrm{W}_a/\mathrm{W}_0$ data for the α-Si nanodisk.

**Optical mode**	**Resonant wavelength (eigenmode analysis)**	**Q-factor (eigen-mode analysis)**	**Resonant wavelength (SNOM probe)**	**Q-factor (SNOM probe)**
TE^{ee}_{41} **(WGM)**	**674 nm**	**120**	**670 nm**	**68**
TE^{eo}_{13} **(CM)**	**686 nm**	**23.5**	**685 nm**	**24**

Tab. S2: Comparison of Q-factor and resonant wavelength of optical modes calculated by eigenmode analysis and SNOM W_a/W_0 data for the α-Si nanosquare.

Optical mode	Resonant wavelength (eigenmode analysis)	Q-factor (eigenmode analysis)	Resonant wavelength (SNOM probe)	Q-factor (SNOM probe)
TE_{41}^{ee} (WGM)	709 nm	58	704 nm	45
TE_{43}^{eo} (CM)	658 nm	110	655 nm	62
TE_{33}^{oo} (CM)	724 nm	133	719 nm	52
TE_{41}^{eo} (CM)	714 nm	20	690 nm	21
TE_{34}^{oe} (CM)	658 nm	110	655 nm	43

Tab. S3: Comparison of Q-factor and resonant wavelength of optical modes calculated by eigenmode analysis and SNOM W_a/W_0 data for the α-Si nanotriangle.

Optical mode	Resonant wavelength (eigenmode analysis)	Q-factor (eigenmode analysis)	Resonant wavelength (SNOM probe)	Q-factor (SNOM probe)
TM_{42}^{e} (CM)	635 nm	23.5	632 nm	21
TM_{32}^{e} (CM)	not possible to calculate due to spectral overlapping and low Q	not possible to calculate due to spectral overlapping and low Q	695 nm	15
TE_{21}^{e} (CM)	710 nm	22	710 nm	18
TE_{32}^{o} (CM)	645 nm	28	642 nm	24
TE_{21}^{o} (CM)	711 nm	23	710 nm	19

6 FDTD simulation of extinction and electric field localization spectra of the single nanodisk, –square, and –triangle under plane wave excitation

The extinction cross-section spectrum (σ_{ext}) of the individual nanodisk, -square, and -triangle on the glass substrate was simulated by Lumerical Solutions FDTD using a total-field scattered-field (TFSF) source under normal illumination incident from the antenna side (see illustration in Figure S3(a) and (d)). The extinction cross-section (black curves in Figure S3(a-d)) was calculated as a sum of the absorption (σ_{abs}) and scattering (σ_{scat}) cross-section. The excitation of optical modes in nanocavities results in maxima of W_a/W_0 (blue curves in Figure S3(a-d)) and an asymmetric Fano lineshape in the extinction cross-section spectra. In comparison with the excitation of optical modes by the SNOM probe, the normal incidence illumination in combination with the symmetry of the plane wave source allows coupling with only specific nanocavities modes. When the polarization is directed along the $y-$axis, the plane wave source possesses even symmetry with respect to the σ_x-plane (perpendicular to the $x-$axis) and odd symmetry with respect to the σ_y-plane (perpendicular to the $y-$axis). Plane wave illumination of the nanodisk (Figure S3(a)) leads to the excitation of the TE_{13}^{eo} mode with an even symmetry with respect to the σ_x-plane and odd symmetry with respect to the σ_y-plane. Whereas the excitation of the TE_{51}^{eo} and TE_{31}^{eo} WGMs (excited by the SNOM probe, see Figure 1(f) in the main text) with the same even/odd symmetry cannot be observed due to the low coupling efficiency in comparison with TE_{13}^{eo} mode. The excitation of TE_{41}^{ee} WGM (Figure 1(f)

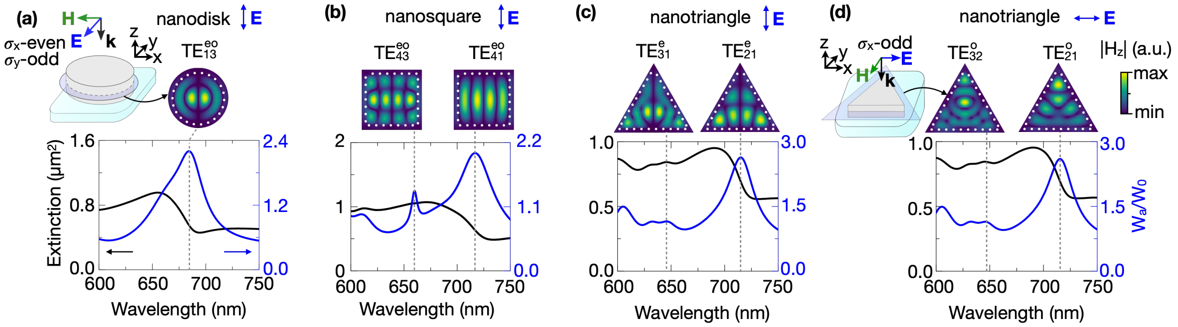

Fig. S3: The simulated electric field localization spectra W_a/W_0 (blue curves) and extinction spectra (black curves) inside the α-Si nanodisk (a), –square (b), and –triangle (c,d) under plane wave illumination at normal incidence. The top row shows the $|H_z|$ distribution of the excited optical modes. The blue arrow indicates the polarization of the incident light. Sketches in (a) and (d) illustrate the scheme of the simulations.

in the main text) with even parity with respect to both symmetry planes is symmetry forbidden by the plane wave at normal illumination. In Table S4, we summarized the optical modes in the nanodisk, which are excited by the SNOM probe and plane wave source. For the nanosquare (Figure S3(b)), TE^{eo}_{43} and TE^{eo}_{41} CMs with σ_x-even and σ_y-odd symmetry are excited. However, the excitation of the TE^{oo}_{33} CM and TE^{ee}_{41} WGM is symmetry forbidden (see Table S5 for a full overview). In the case of the nanotriangle illuminated by $y-$polarized light (Figure S3(c)), the excitation of TE^{e}_{31} and TE^{e}_{21} modes with σ_x-even symmetry is possible. Comparing the SNOM probe excitation and plane wave illumination, the SNOM probe excites σ_x-even symmetry TM^{e}_{42}, TM^{e}_{32}, TE^{e}_{40}, and TE^{e}_{21} modes (see Table S6), while the TE^{e}_{31} mode is not observed due to the higher coupling efficiency of the SNOM probe with other CMs of the nanotriangle. When the polarization of the plane wave source is changed to be oriented along the $x-$axis (panel (d)), the parity of the source also changes to odd with respect to the σ_x-plane. This leads to the excitation of σ_x-odd TE^{o}_{32} and TE^{o}_{21} modes. The SNOM probe excitation also allows visualization of these modes along with TE^{o}_{31}, TE^{o}_{30}, modes with σ_x-odd symmetry (see Table S7).

Tab. S4: The list of optical modes excited in the α-Si nanodisk by SNOM probe and plane wave (PW) source at normal incidence

**Excited modes by SNOM probe**	TE^{eo}_{51}	TE^{ee}_{41}	TE^{eo}_{31}	TE^{eo}_{13}
**Excited modes by PW at normal incidence**	×	×	×	✓
**Symmetry allowed modes for PW excitation at normal incidence**	✓	×	✓	✓
**Modes not excited by the PW source at normal incidence due to low coupling efficiency**	✓	×	✓	×

Tab. S5: The list of optical modes excited in the α-Si nanosquare by SNOM probe and plane wave (PW) source at normal incidence.

**Excited modes by SNOM probe**	TE^{eo}_{43}	TE^{ee}_{41} **(WGM)**	TE^{oe}_{34}	TE^{oo}_{33}	TE^{eo}_{41}
**Excited modes by PW at normal incidence**	✓	×	✓	×	✓
**Symmetry allowed modes for PW excitation at normal incidence**	✓	×	✓	×	✓
**Modes not excited by the PW source at normal incidence due to low coupling efficiency**	×	×	×	×	×

Tab. S6: The list of optical modes excited in the α-Si nanotriangle ($y-$polarization) by SNOM probe and plane wave (PW) source at normal incidence ($y-$polarization).

Excited modes by SNOM probe	TM^e_{42}	TM^e_{32}	TE^e_{21}	TE^e_{40}
Excited modes by PW at normal incidence	×	×	✓	×
Symmetry allowed modes for PW excitation at normal incidence	✓	✓	✓	✓
Modes not excited by the PW source at normal incidence due to low coupling efficiency	✓	✓	×	✓

Tab. S7: The list of optical modes excited in the α-Si nanotriangle ($x-$polarization) by SNOM probe and plane wave source (PW) at the normal incidence ($x-$polarization).

Excited modes by SNOM probe	TE^o_{32}	TE^o_{21}	TE^o_{31}	TE^o_{30}
Excited modes by PW at normal incidence	✓	✓	×	×
Symmetry allowed modes for PW excitation at normal incidence	✓	✓	✓	✓
Modes not excited by the PW source at normal incidence due to low coupling efficiency	×	×	×	×

References

[1] A. Y. Frolov, N. Verellen, J. Li, X. Zheng, H. Paddubrouskaya, D. Denkova, M. R. Shcherbakov, G. A. E. Vandenbosch, V. I. Panov, P. Van Dorpe, A. A. Fedyanin, and V. V. Moshchalkov. Near-field mapping of optical fabry–perot modes in all-dielectric nanoantennas. *Nano Lett.*, 17(12):7629–7637, 12 2017.

[2] Witec wissenschaftliche instrumente und technologie gmbh available at:. URL http://www.witec.de.

[3] Lumerical fdtd solutions. available at:. URL http://www.lumerical.com.

[4] A. Alex-Amor, G. Valerio, F. Ghasemifard, F. Mesa, P. Padilla, J. M. Fernández-González, and O. Quevedo-Teruel. Wave propagation in periodic metallic structures with equilateral triangular holes. *Appl. Sci.*, 10(5), 2020.

[5] C. Wang. Exact solution of equilateral triangular waveguide. *Electron. Lett.*, 46(13):925–927, 2010.
